# Effects of Therapeutic Hypothermia Combined with Other Neuroprotective Strategies on Ischemic Stroke: Review of Evidence

**DOI:** 10.14336/AD.2017.0628

**Published:** 2018-06-01

**Authors:** Zheng Zhang, Linlei Zhang, Yuchuan Ding, Zhao Han, Xunming Ji

**Affiliations:** ^1^Department of Neurosurgery, Xuanwu Hospital, Capital Medical University, Beijing, China; ^2^Department of Neurology, the First Affiliated Hospital, Wenzhou Medical University, Wenzhou, China; ^3^Department of Neurology, the Second Affiliated Hospital, Wenzhou Medical University, Wenzhou, China; ^4^Department of Neurological Surgery, Wayne State University School of Medicine, Detroit, MI, USA; ^5^China-America Institute of Neuroscience, Xuanwu Hospital, Capital Medical University, Beijing, China

**Keywords:** ischemic stroke, neuroprotection, therapeutic hypothermia, combination therapy

## Abstract

Ischemic stroke is a major cause of death and disability globally, and its incidence is increasing. The only treatment approved by the US Food and Drug Administration for acute ischemic stroke is thrombolytic treatment with recombinant tissue plasminogen activator. As an alternative, therapeutic hypothermia has shown excellent potential in preclinical and small clinical studies, but it has largely failed in large clinical studies. This has led clinicians to explore the combination of therapeutic hypothermia with other neuroprotective strategies. This review examines preclinical and clinical progress towards developing highly effective combination therapy involving hypothermia for stroke patients.

Hypothermia is a remarkable neuroprotectant and can mitigate brain injury induced by stroke, trauma, cardiac arrest or hypoxic-ischemic encephalopathy in newborns [1-3]. Yet how hypothermia protects the brain and what are the optimal conditions for protection are unclear. Studies suggest that initiating hypothermia as soon as possible after ischemia is better [4, 5], and that longer periods of hypothermia are superior to shorter periods [6]. Hypothermia appears to give better results when reperfusion occurs, though it is unclear whether hypothermia provides benefit in the case of permanent ischemia [5, 7]. The depth and duration of hypothermia strongly influence the risk of complications such as infection [8] and arrhythmias [9]. These adverse events and the lack of optimized procedures have limited the application of hypothermia in the clinic.

To overcome these limitations, preclinical and clinical studies over the last few decades have been exploring the combination of hypothermia with a second neuroprotective strategy (Table 1). Such combination therapy is intended to prolong the therapeutic window of both treatments while reducing or eliminating adverse effects, thereby enhancing the magnitude and duration of neuroprotection. The present review discusses various combination approaches and summarizes their preclinical and clinical application in ischemic stroke.

**Table 1:** summary of the second neuroprotective strategies combined with hypothermia in ischemic stroke.

Main Function of combined strategies	Combined strategies	Specified treatment
Reduce energy consumption	Anesthetics	Methohexital Thiopentone sodium Xenon Dexmedetomidine
	Psychotropic agents	Chlorpromazine and promethazine
Suppress calcium overload	NMDA receptor antagonists Ryanodine receptor inhibitor	Dextromethorphan MK-801 Delfotel Magnesium with or without tirilazad Dantrolene
Increase blood supply	Reperfusion Vessel dilator Induce arteriogenesis	t-PA Intra-arterial recanalization Statin Granulocyte colony stimulating factor (G-CSF)
Anti-inflammation	Antibiotics	Tacrolimus Minocycline
Anti-oxidative stress	Oxidative stress scavengers	Edaravone Mannitol
Repair damaged cells	Biosynthesis of cell component	Citicoline
Increase oxygen supply	Oxygen	Normobaric oxygen Hyperbaric oxygen
Reduce intracranial pressure	-	Decompressive craniectomy
Anti-apoptosis	Anti-apoptosis protein	FNK protein
	Gene of anti-apoptosis protein	Bcl-2 gene
Multiple protection	-	Caffeinol (caffeine and ethanol) Insulin-like growth factor-1 (IGF-1) Brain-derived neurotrophic factor (BDNF) Albumin

## 1. Combination therapy involving antagonists of the *n*-methyl-d-aspartate (nmda) receptor

### Therapies aiming at reducing exitotoxicity (preclinical evidence)

In this approach, hypothermia is combined with the administration of NMDA receptor antagonists in order to reduce excitotoxicity. To date, this approach has been tested in preclinical studies. The approach is based on the fact that the abrupt deprivation of oxygen and energy depolarizes neurons and glial cells, which release large amounts of excitatory amino acids into the synaptic cleft. These amino acids bind to several receptors, one of which is the NMDA receptor, which in response triggers calcium influx that leads to programmed cell death. Not surprisingly, efforts to develop neuroprotective therapies have been focusing on NMDA receptors for decades, and various antagonists have been studied on their own or combined with hypothermia.

The first studies were conducted using the non-competitive antagonists dextromethorphan and MK-801. In one study, animals were exposed to MK-801, hypothermia (33°C) or both and then subjected to permanent middle cerebral artery occlusion [10]. Either MK801 or hypothermia on their own reduces infarct volume, while the combination of both did not show an additive protective effect. In another study, animals were subjected to transient middle cerebral artery occlusion, followed by hypothermia (30°C, 3 h) and later by MK-801 therapy given 3-7 days after ischemia [11]. The combination of post-ischemia hypothermia and delayed MK-801 therapy attenuated neurobehavioral deficits at various follow-up points, and neuroprotection in the CA1 hippocampus was observed histologically up to 2 months after ischemia [12].

It is difficult to understand the mechanism of combination therapies involving hypothermia and non-competitive antagonists such as MK-801, since this compound by itself induces hypothermia [13, 14]. As a result, it is difficult to optimize combination therapy parameters. In addition, non-competitive blockade of NMDA receptors may cause severe adverse effects [16, 17] because low-dose NMDA is necessary for normal neuronal function and survival [15].

Therefore, research has also focused on competitive NMDA receptor antagonists such as selfotel (CGS-19755). Despite being a potent antagonist, this compound did not provide significant neuroprotection in gerbils when administered alone or with hypothermia 30 min after 5-min forebrain ischemic insult [18]. In contrast, the compound helped protect various parts of the brain when administered with hypothermia after a series of three transient ischemic insults lasting 3 min each and delivered 1 h apart [19].

An alternative to exogenous antagonists is to use endogenous antagonists such as magnesium ion (Mg^2+^). Mg^2+^ exerts anti-excitotoxic effects by antagonizing calcium entry via the NMDA receptor [20]. On its own, Mg^2+^ does not confer neuroprotection in transient or permanent stroke, but when combined with hypothermia, it has shown promising benefits in both injury contexts [21, 22]. In fact, the combination of magnesium and hypothermia proved superior to hypothermia alone in an animal model of transient middle cerebral artery occlusion [23]. On the other hand, a study of permanent middle cerebral artery occlusion failed to find a benefit of the combination of Mg^2+^ and hypothermia after ischemic stroke [24].

These precedents suggest that combination therapy shows potential but that further work is needed to optimize when it is delivered relative to the stroke and at what doses. Obtaining optimal neuroprotection will likely require a deeper understanding of how the two components of the combination therapy act separately and together.

## 2. Combination therapy involving reperfusion

### Therapies aiming at increasing brain perfusion (preclinical evidence)

In this approach, hypothermia is combined with recombinant tissue plasminogen activator therapy, which is the only treatment for acute ischemic stroke approved by the US Food and Drug Administration. The rationale behind combining hypothermia and plasminogen activator therapy is that reperfusion helps to rapidly restore oxidative metabolism in surviving cells, resolve cytotoxic edema and reduce levels of excitatory amino acids. On the other hand, reperfusion can trigger NO release and superoxide formation as well as increase permeability of the blood brain barrier (BBB), which can lead to further excitotoxicity and hemorrhagic transformation. Hypothermia induced shortly after reperfusion can reduce NO efflux in the brain and BBB leakiness, minimizing reperfusion injury [25, 26].

One study reported that combining hypothermia and plasminogen activator reduced infarct size as well as the hemorrhagic transformation ratio that plasminogen activator therapy usually increases [27]. Hypothermia also alleviated the adverse effects of plasminogen activator therapy, such as larger infarct volume and brain edema, and it mitigated reperfusion injury caused by matrix metalloproteinase-1 (TIMP-1) and soluble intercellular adhesion molecule-1 (sICAM-1) [28].

Nevertheless, the benefits of this combination therapy are not without controversy. Studies of embolic stroke reported no therapeutic enhancement when the two treatments were combined [29, 30]. Other studies showed that hypothermia yielded neuroprotection in transient ischemia, but it gave mixed results in permanent ischemia, it gave mixed results in permanent ischemia [5, 7]. Those studies suggested that hypothermia may give better results when performed with vascular recanalization.

These results suggest a somewhat inconsistent picture of therapeutic benefits of combination therapy, highlighting the need for more detailed mechanistic insights.

### Therapies aiming at increasing brain perfusion (clinical evidence)

On the basis of the strong benefits reported for thrombolysis or hypothermia on their own, clinical trials have explored the safety and efficacy of combining the two for treating acute ischemic stroke patients. The ICTuS-L trial recruited acute ischemic stroke patients within 6h of symptom onset and administered intravenous thrombolysis with or without 24-h endovascular cooling (33°C) [31]. Based on assessment using the modified Rankin Scale (mRS), combination therapy was not superior to thrombolysis alone. In another clinical trial, thrombolysis was administered to ischemic patients within 6 h of symptom onset with or without 1-h local hypothermia therapy (32-34°C) on the surface of the lesion side. Once again, combination therapy was not superior to thrombolysis alone, based on assessments using the National Institutes of Health Stroke Scale (NIHSS) score or the Barthel Index (BI) [32].

Therapeutic benefit of combination therapy may depend on the relative timing of the procedures: performing hypothermia after thrombolysis has been shown to reduce edema and hemorrhagic transformation [33] as well as improve clinical outcomes [34]. Further work is needed to clarify the influence of timing. At the very least, the available evidence clearly shows that the combination of hypothermia and thrombolysis is safe and feasible in acute ischemic patients. These trials have also demonstrated the safety of intra-arterial hypothermia, which can rapidly reduce brain temperature significantly while avoiding adverse effects associated with systemic cooling.

Endovascular mechanical thrombectomy (intra-arterial recanalization) is an alternative to plasminogen activator therapy that has significantly improved the success rate of stroke treatment in recent years [35, 36]. One clinical study showed good results when acute ischemic stroke patients were subjected, within 8 h after stroke onset, to intra-arterial hypothermia followed by intra-arterial recanalization. Clots were mechanically removed using a stent retriever inserted through an angiographic catheter [37]. The combination therapy was performed without technical errors in all 26 patients, without obvious complications related to the hypothermia.

These clinical studies show the feasibility and safety of combining hypothermia with plasminogen activator therapy or intra-arterial recanalization in acute stroke patients. They also highlight the feasibility of intra-arterial hypothermia as a potentially safer alternative to systemic cooling. Further work is needed to determine whether such combination therapies are superior to thrombolysis or recanalization on their own.

## 3. Combination therapy involving anti-inflammatory factors

### Therapies aiming at anti-inflammation (preclinical evidence)

This approach has been tested so far only in preclinical studies. It is well known that neuroinflammation is a key element in the ischemic cascade after cerebral ischemia, and that it leads to the damage and death of neurons in the subacute phase. This death and especially the release of necrotic cell debris triggers inflammation, strongly activating phagocytic cells [38]. Induction of mild-to-moderate hypothermia in the ischemic brain inhibits local inflammation and is believed to contribute to neuroprotection [6, 39]. In addition, FK506 (Tacrolimus), an agent that suppresses the release of inflammatory cytokines and decreases NO synthase expression, was found to protect animals against ischemic injury [40, 41], though its therapeutic window was shorter than 2 h, limiting its clinical application. Combining FK506 with hypothermia (35°C) prolonged the therapeutic window and led to smaller infarct volume and edema than either treatment on its own [42].

The anti-inflammatory effects of minocycline, a second-generation, semi-synthetic tetracycline, are thought to contribute to its ability to protect neurons from ischemic injury in preclinical models [43]. Various studies suggest that it may act by inhibiting the activity of inducible nitric oxide synthase (iNOS) [44] and matrix metalloproteinase (MMPs) [45], inhibiting the activation of caspase-1 and -3 [46], and enhancing the effects of Bcl-2 [47]. One study involving transient middle cerebral artery occlusion found a small, albeit non-significant, increase in therapeutic benefit when minocycline was combined with hypothermia (33°C, 4 h) relative to either treatment on its own [25]. Further work is required to explore this combination therapy since a study involving permanent middle cerebral artery occlusion found no such additive effect [48]. Delaying the use of minocycline can still provide neuroprotection, but combining delayed minocycline with delayed hypothermia (34-35°C) did not enhance protective effects [49]. Clearly the research into combination therapy involving hypothermia and anti-inflammatory treatments is in very early stages.

## 4. Combination therapy involving statins

### Therapies aiming at increasing brain perfusion (preclinical evidence)

This approach aims to increase brain perfusion in order to protect neurons from ischemic injury, and so far, it has been evaluated only in preclinical studies. Statins up-regulate endothelial nitric oxide (NO) synthase (eNOS) and are recommended for treating patients with atherosclerotic ischemia [50]. NO is a major vasodilator produced by cerebrovascular endothelium to maintain sufficient cerebral blood flow and normal brain function [51]. Combining atorvastatin with hypothermia (32-33°C, 2 h) led to smaller infarct volume than either therapy separately and it expanded the therapeutic window of hypothermia from 3 to 6 h after ischemia [52]. This promising approach should be investigated in greater detail in preclinical studies and ultimately in clinical trials.

## 5. Combination therapy involving oxidative stress scavengers

### Therapies aiming at scavenging free radical (preclinical evidence)

This approach, which has been tested so far only in preclinical studies, aims to scavenge excess free radicals produced when cerebral ischemia and reperfusion injury perturb the balance between free radical production and degradation [53]. Since 2002, for example, the free radical scavenger edaravone (3-methyl-1-phenyl-2-pyrazolin-5-one) has been approved for the treatment of stroke in Asia [54]. This scavenger is believed to interact with peroxyl and hydroxyl radicals, creating a radical intermediate that forms stable oxidation products [55, 56]. The combination of edaravone and hypothermia (35°C) was found to significantly reduce edema and infarct volume in rats subjected to transient focal cerebral ischemia [57]; in contrast, edavarone on its own reduced edema, while hypothermia on its own showed no neuroprotective effect.

Mannitol is a free-radical scavenger also used clinically as a dehydrating agent because it can reduce intracranial pressure by creating an osmotic pressure gradient [58]. Combining mannitol and hypothermia (32-34°C) that was begun 1 h before permanent middle cerebral artery occlusion and continued for 2 h afterwards led to infarct volume similar to that with hypothermia alone [59]. Similarly, combining mannitol with hypothermia (33°C) lasting 1 h through the ischemic process did not enhance the therapeutic effects of hypothermia in a rat model of transient ischemia [60].

## 6. Combination therapy involving citicoline

### Therapies aiming at biosynthesis of cell component (preclinical evidence)

This approach, which has been evaluated so far only in preclinical studies, takes advantage of the ability of citicoline to mimic cytidine-5’-diphosphocholine, which is an intermediate in the generation of phosphatidylcholine and is essential for the biosynthesis of membrane phospholipids [61]. Citicoline restores the activity of mitochondrial ATPase and membrane Na^+^/K^+^ ATPase, and it inhibits the activation of phospholipase A2, the formation of free radicals and the release of free fatty acids [62, 63]. Citicoline can influence the ischemia cascade at different levels to produce neuroprotective effects [64]. Combining citicoline with hypothermia (34°C) was more effective than either therapy alone at suppressing apoptosis [65].

## 7. Combination therapy involving psychotropic agents

### Therapies based on multiple mechanisms (preclinical evidence)

The combination of low-dose caffeine and ethanol, termed caffeinol, can effectively reduce brain damage in rodent models of focal cerebral ischemia [66], perhaps by stimulating adenosine-mediated transduction pathways that inhibit gamma-aminobutyric acid (GABA) and NMDA receptors [67]. The neuroprotective effect of caffeinol can be enhanced by combining it with hypothermia (35°C) in animal models of transient occlusion of the middle cerebral artery [68]. The therapeutic window needs to be determined carefully, since administering caffeinol daily for two weeks before ischemic stroke onset eliminated its neuroprotective effects.

### Therapies based on multiple mechanisms (clinical evidence)

In addition to preclinical studies, the combination of caffeinol and hypothermia has been explored in patients. In one study in which caffeinol, tissue plasminogen activator and hypothermia (33-35°C) induced from 5 to 24 h after stroke onset were given to patients with acute ischemic stroke, caffeinol did not enhance the effects of plasminogen activator [69].

### Therapies aim at reducing energy consumption (preclinical evidence)

Phenothiazines are among the oldest synthetic anti-psychotic drugs. They block dopaminergic receptors to induce neuroleptic effects, but they also reduce energy consumption by inhibiting calmodulin, protein kinase C, and P-glycoprotein transport, suppressing cell proliferation [70, 71]. Combining chlorpromazine and promethazine (both 1? mg/kg) with 5-min hypothermia (35°C) at the initiation of reperfusion following 2-h occlusion of the middle cerebral artery led to smaller infarct volume and long-term neurological deficit [72]. In contrast, neither treatment on its own showed neuroprotection.

## 8. Combination therapy involving anesthetic-related agents

### Therapies aiming at reducing energy consumption (preclinical evidence)

Numerous preclinical studies have explored the combination of hypothermia with barbiturates and volatile anesthetics, which on their own have been shown to protect against cerebral damage in animal models of focal ischemia [73, 74]. These drugs may exert protective effects by reducing cellular energy requirements, improving blood flow into ischemic brain tissue, and counteracting oxidative stress and excitotoxicity [75, 76].

Methohexital administered 30 min before stroke reduced infarct volume and neurological score in a preclinical study, and these benefits were not enhanced by adding hypothermia [77]. In a study involving cortical neuron cultures exposed to prolonged hypoxia (24-48 h) [78], the combination of thiopentone sodium with mild hypothermia (32°C) or deep hypothermia (22°C) provided greater neuroprotection than either therapy on its own. Xenon, a potent anesthetic, has shown neuroprotective effects in adult rats subjected to transient brain ischemia [79]. The mechanisms underlying this neuroprotection may involve interaction with NMDA receptors, down-regulation of cytosolic pro-apoptotic Bax protein, up-regulation of Bcl-xL expression and increased phosphorylation of transcription factor cAMP-response element binding protein [80, 81]. Xenon and hypothermia have shown additive therapeutic effects in hypoxia-ischemia models [82, 83], as well as in a model of transient middle cerebral artery occlusion [84]. Administering the α2 agonist dexmedetomidine before ischemia significantly reduced subsequent levels of plasma catecholamines and decreased neurological comorbidities [85], potentially by reducing oxidative stress as well as inhibiting inflammation [86] and apoptosis [87]. Combining dexmedetomidine (given 30 min before ischemia) with hypothermia (35°C) from 1 h before ischemia until 1 h after reperfusion onset showed no additional benefit relative to either therapy on its own [88].

The anesthetic dantrolene, an inhibitor of ryanodine receptors, shows good preclinical promise for enhancing the therapeutic benefits of hypothermia after stroke. It blocks the release of Ca^2+^ stores from endoplasmic reticulum, protecting neurons from oxygen-glucose deprivation toxicity [89] and apoptosis [90]. Dantrolene can also accelerate body temperature cooling [91] and reduce incidence of shivering and shivering thermogenesis [92], both common side effects of hypothermia. For these reasons, dantrolene has been approved for treating malignant hyperthermia after anesthesia. One study found that combining dantrolene with hypothermia (33°C) helped protect cerebral cortex neurons from oxygen-glucose deprivation, increasing neuronal survival and mitochondrial membrane potential as well as reducing DNA fragmentation and apoptosis [93].

## 9. Combination therapy involving normo- and hyperbaric oxygen

### Therapies aiming at increasing oxygen supply (preclinical evidence)

One of the initiators of ischemic pathophysiology is shortage of oxygen to the brain. Normobaric hyperoxia (NBO), induced by breathing air containing 21-100% oxygen at a pressure of 1 absolute atmosphere, effectively reduced infarct volume and neurological deficits in rodents following acute ischemic stroke [94, 95]. NBO can also reduce BBB permeability and extend the therapeutic window of reperfusion therapy [96, 97]. In animals subjected to 1-h occlusion of the middle cerebral artery, the combination of tissue plasminogen activator-induced reperfusion, NBO (60%) and 3-h hypothermia (33°C) starting 1 h after infarction reduced infarct volume, neurological deficit and production of reactive oxygen species [98]. These effects were associated with an increase in pyruvate dehydrogenase activity and protein expression. Combining NBO with hypothermia reduced reperfusion injury by modulating NADPH oxidase activity and attenuating hyperglycolysis [99, 100].

Hyperbaric oxygen (HBO) may provide superior neuroprotection than NBO during transient and permanent cerebral ischemia [101, 102]. At the same time, HBO has been shown to induce oxidative stress in animal studies, likely reflecting prolonged exposure to highly concentrated oxygen [103, 104]. Performing HBO (2 absolute atmospheres, 100% oxygen, 60 min) during rewarming from moderate hypothermia (31°C, 60 min) preserved CA1 pyramidal neurons better than hypothermia alone [105].

## 10. Combination therapy involving granulocyte colony-stimulating factor

### Therapies aiming at inducing arteriogenesis and brain perfusion (preclinical evidence)

Hematopoietic cytokines, by binding receptors on the membranes of neurons and glial cells in the central nervous system, stimulate intracellular signaling pathways that can help protect against injury and/or support neurogenesis [106, 107]. The cytokine granulocyte colony-stimulating factor (G-CSF) stimulates growth and differentiation of hematopoietic cells and is clinically used to treat chemotherapy-induced neutropenia [108]. It induces arteriogenesis in the hypo-perfused brain of rats and mice, restores cerebral blood flow, and mitigates stroke severity [109-112]. G-CSF may exert neuroprotective effects by mobilizing stem cells, preventing apoptosis, promoting neurogenesis, and reducing inflammation [113, 114]. Combining G-CSF with mild, 30-min hypothermia (33.5-35°C) initiated at reperfusion significantly reduced mortality, edema and neurological deficit after transient middle cerebral artery occlusion [115].

## 11. Combination therapy involving growth factors

### Therapies based on multiple pretection (preclinical evidence)

Insulin-like growth factors (IGFs) are peptide hormones with significant structural homology to insulin. IGF-1 is a potent cardiomyocyte growth and survival factor [116], which can regulate cell proliferation and inhibit cell apoptosis and necrosis after ischemic stroke [117-119]. The combination of IGF-1 and hypothermia was tested in a study in which rats were subjected to global ischemia lasting 8 min. Afterwards, animals were treated with hypothermia (32°C, 4 h) and/or IGF-1 (0.6 U/kg), then spatial memory was assessed at 21 days and CA1 neurons were counted on day 7 or 28 [120]. The combination of both treatments starting at the onset of reperfusion preserved CA1 structure and memory at 28 days better than either treatment on its own. On the contrary, another study indicated that after cerebral ischemia lasting 30 min, white matter damage was similar between animals treated with the combination of IGF-1 (3 µg, ventricularly) and hypothermia (30-33°C) from 5.5 h after ischemia until 72 h after ischemia, and animals treated with hypothermia alone, based on immunohistochemistry and numbers of caspase 3-positive cells [121].

## 12. Combination therapy involving brain-derived neurotrophic factor (bdnf)

### Therapies based on multiple pretection (preclinical evidence)

Brain-derived neurotrophic factor (BDNF) plays an important role in proliferation, differentiation, maintenance, plasticity, survival and neurite extension in the central and peripheral nervous systems [122]. BDNF administered intraventricularly or intravenously can protect the brain from transient or permanent ischemic insult [123, 124]. It may exert these effects by countering the activity of Bax and Bcl-2 proteins within the ischemic penumbra [125], inhibiting glutamate and NO neurotoxicity [126] and reversing NMDA-induced inactivation of protein kinase C [127]. In a model of the hyperacute phase of permanent middle cerebral artery occlusion, combining BDNF [300 mg/(kg/h), 2 h) with hypothermia (33°C) at 30 min after occlusion onset reduced post-ischemic glutamate concentration and infarct volume [128].

## 13. Combination therapy involving magnesium and tirilazad

### Therapies aiming at reducing exitotoxicity (preclinical evidence)

Mg^2+^ is a non-competitive endogenous NMDA antagonist [10], and tirilazad (U-74006F) is a non-glucocorticoid, 21-aminosteroid that inhibits lipid peroxidation. Tirilazad is thought to inhibit iron-dependent lipid peroxidation within membranes through free-radical scavenging of lipid peroxyl and hydroxyl groups. This reduces the formation of hydroxyl radicals and maintains the levels of endogenous antioxidants [129]. Administering tirilazad before ischemia reduced stroke lesion size, neuronal necrosis, brain injury, and cerebral edema in rodents exposed to permanent or transient ischemia, while administering it after ischemia provided no protection [130, 131]. The combination of Mg^2+^ and tirilazad given pre-ischemia resulted in better neurologic function and smaller infarct volume [132]. Combining these two agents with 3-h hypothermia (33°C) starting 20 min before transient ischemia further decreased subsequent infarct volume and improved electroencephalography amplitude and neurological function [133]. The same researchers also showed that the triple combination of Mg^2+^, tirilazad and hypothermia gave better results than the combination of nimodipine, mannitol, dexamethasone, and methohexital [134]. At least in a model of transient infarction, the triple combination of Mg^2+^, tirilazad and hypothermia showed potent neuroprotection when administered before ischemia onset or up to 3 h afterwards [135].

## 14. Combination therapy involving albumin

### Therapies based on multiple pretection (preclinical evidence)

This approach, which has been evaluated only in preclinical studies, is based on the ability of albumin to inhibit endothelial apoptosis and to act as an antioxidant [136]. It also causes hemodilution, helps maintain microvascular permeability and regulates the enzyme pyruvate dehydrogenase. Administering low-dose cold albumin (0.5 g/kg, 0°C) via the middle cerebral artery to induce 45-min local hypothermia (30°C) in the ischemic lesion led to smaller infarct volume and better neurological outcomes than normothermia albumin [137].

## 15. Combination therapy involving decompressive craniectomy

### Therapies aiming at decreasing intracranial pressure (preclinical evidence)

Decompressive craniectomy, in which part of the skull is removed and the dura is opened, can reduce intracranial pressure; edema is a lethal complication of malignant supratentorial stroke, and it leads to high mortality [138]. This surgical procedure is superior to conservative treatment at reducing mortality [139]. While hypothermia on its own can also reduce intracranial pressure, the pressure can rebound during or after rewarming and cause lethal herniation [140]. Combining 5-h hypothermia (32°C) and decompressive craniectomy in rat models of permanent middle cerebral artery occlusion significantly reduced infarct volume and improved neurological outcome [141]. Applying this combination therapy even up to 6 h after stroke gave better results than either therapy on its own, based on infarct volume, neurological score and BBB integrity [142].

### Therapies aiming at decreasing intracranial pressure (clinical evidence)

The combination of hypothermia and decompressive craniectomy has also been evaluated in clinical trials. The combination of surgery and hypothermia (35°C) induced immediately afterward led to better clinical outcomes than surgery alone in patients with malignant supratentorial stroke [143].

## 16. Combination therapy involving protein

### Therapies aiming at anti-apoptosis (preclinical evidence)

This approach, which has been evaluated only in preclinical studies, takes advantage of the fact that Bax promotes apoptosis, whereas Bcl-2 and Bcl-xL inhibit apoptosis by blocking the translocation of cytochrome C. Substituting three amino acids in Bcl-xL enhances the anti-apoptotic activity, and the resulting variant, called FNK, can be fused to the protein transduction domain of HIV/Tat protein and efficiently introduced into cells [144]. In cell culture studies, FNK provided greater protection than Bcl-xL against oxidative stress, calcium ionophores, growth factor withdrawal, heat, or treatment with anti-Fas agents, cell cycle inhibitors or a protein kinase inhibitor [145]. FNK has been shown to ameliorate ischemic damage *in vivo* and *in vitro* [146]. Combining FNK with mild, 2-h hypothermia (35°C) starting from ischemia onset until reperfusion enhanced neuroprotection in rats following transient middle cerebral artery occlusion, potentially by inhibiting pro-apoptotic pathways mediated by caspase-12, and not by promoting anti-apoptotic pathways mediated by Bcl-2 [147].

## 17. Gene-based combination therapy

### Therapies aiming at anti-apoptosis (preclinical evidence)

The rationale of gene therapy is to insert genes encoding neuroprotective proteins into neurons to promote their survival against cerebral insults [148]. Such therapy can lead to protective effects only after hours or even days. Administering simplex virus (HSV) vector expressing Bcl-2 led to neuroprotection via Bcl-2 overexpression when it was given 1.5 h after stroke but not 5 h after stroke [149]. To prolong the therapeutic window of gene therapy, researchers have combined it with hypothermia. Indeed, in one study, combining Bcl-2 gene treatment with hypothermia (33°C) induced after ischemia prolonged the therapeutic window of Bcl-2 oxerexpression from 1.5 h to 5 h, and it blocked the release of cytochrome C up to 48 h after ischemia [150].

## Conclusions

The combination of diverse neuroprotective strategies with hypothermia has been extensively investigated for the alleviation of ischemic injury arising from cardiac arrest, hypoxic-ischemic encephalopathy or spinal cord impairment. The literature applying such combination therapy to focal stroke is more limited. In addition, studies of such combination therapy often report inconsistent results about the efficacy for treating stroke (Table 2). Several differences among studies may help explain these discrepancies, such as how stroke was modeled, when treatment was initiated relative to stroke, how long treatment lasted, and what was the depth of cooling. As far as neuroprotective drugs are concerned alone, heterogeneity of human stroke and lack of methodological agreement between preclinical and clinical studies may lead to failure of translating experimental success to clinical. Another potential problem is that drug metabolism may differ at cooler temperatures from at normal body temperature. The finding by several studies that combination therapy failed to improve on the results of monotherapy may reflect in some cases a “ceiling effect”: the monotherapy achieved the optimal efficacy, such that additional benefit from the other therapy was undetectable.

**Table 2:** Summary of outcomes of neuroprotective treatments combined with therapeutic hypothermia in ischemic stroke.

Reference	Permanent(P)/ Transient (T) Ischemia	Temperature degree(°C)	Combined Treatment	Aim of study	Yes/No
Animal					
[10]	P	33	Mk-801	Enhanced effect	No
[11]	T	30	Mk-801	Effective	Yes
[12]	T	30	Mk-801	Enhanced effect	Yes
[18]	T	34	Selfotel	Enhanced effect	No
[19]	Repetitive	34	Selfotel	Enhanced effect	Yes
[21]	T	35.4	Magnesium	Enhanced effect	Yes
[22]	P	35	Magnesium	Enhanced effect	Yes
[23]	T	33-34	Magnesium	Enhanced effect	Yes
[24]	P	35	Magnesium	Enhanced effect	No
[27]	T	33	t-PA	Reduce the side effect of t-PA	Yes
[28]	T	34	t-PA	Reduce the side effect of t-PA	Yes
[29]	T	32	Delayed t-PA	Enhanced effect	No
[30]	T	33	t-PA	Enhanced effect	No
[42]	T	35	Tacrolimus	Enhancec/expand time window of tacrolimus	Yes
[52]	T	32-33	Atorvastatin	Enhanced effect/ expand time window of hypothermia	Yes
[57]	T	35	Edaravone	Enhanced effect	Yes
[65]	T	34	Citicoline	Enhanced effect	Yes
[25]	T	33	Minocycline	Enhanced effect	Yes
[48]	P	34	Minocycline	Enhanced effect	No
[49]	P	34-35	Minocycline	Enhanced effect	No
[69]	T	35	Caffeinol	Enhanced effect	Yes
[72]	T	35	Chlorpromazine and Promethazine	Enhanced effect	Yes
[77]	T	33	Methohexital	Enhanced effect	No
[84]	T	36	Xenon	Enhanced effect	Yes
[88]	Incomplete	35	Dexmedetomidine	Enhanced effect	No
[98]	T	33	t-PA and Normobaric Oxygen (NBO)	Enhanced effect	Yes
[99] [105]	T -	33 31	t-PA And normobaric oxygen (NBO) Hyperbaric oxygenation (HBO2)	Enhanced effect Enhanced effect	Yes Yes
[100]	T	33	t-PA and normobaric oxygen (NBO)	reduce the side effect of t-PA	Yes
[115]	T	33.5-35	Granulocyte-Macrophage Colony-Stimulating Factor (G-CSF)	Enhanced effect	Yes
[120]	T	32	Insulin-Like Growth Factors -1(IGF-1)	Enhanced effect	Yes
[121]	T	30-33	Insulin-Like Growth Factors -1(IGF-1)	Enhanced effect	No
[128]	P	33	Brain-Derived Neurotrophic Factor (BDNF)	Enhanced effect	Yes
[133]	T	33	Magnesium and Tirilazad	Enhanced effect	Yes
[134]	T	33	Magnesium and Tirilazad	Effective	Yes
[135]	T	33	Magnesium and Tirilazad	Enhanced effect	Yes
[59]	P	32-34	Mannitol	Enhanced effect	No
[60]	T	33	Mannitol	Enhanced effect	No
[137]	T	30-31	Albumin	Enhanced effect	Yes
[141]	P	32	Decompressive Craniectomy	Enhanced effect	Yes
[142]	P	29-31	Decompressive Craniectomy	Enhanced effect	Yes
[147]	T	35	PTD-FNK	Enhanced effect	Yes
[150]	T	33	Gene of Bcl-2	Expand time window/Enhanced effect	Yes
Neuronal culture					
[78]	-	22/32	Thiopentone Sodium (TPS)	Enhanced effect	Yes
[93]	-	33	Dantrolene	Enhanced effect	Yes
Clinical					
[31]	T	33	t-PA	Enhanced effect	No
[32]	T	32-34	t-PA	Enhanced effect	No
[33]	T	<35.5	t-PA	Feasible/improve outcome	Yes
[34]	T	34.5	t-PA	Reduce the side effect of tPA	Yes
[37]	T	decrease 2 in brain	Intra-Arterial Recanalization	Feasible and safe	Yes
[69]	T	33-35	Caffeinol and T-PA	Feasible	Yes
[143]	P	35	Decompressive Craniectomy	Enhanced effect	Yes

An important consideration when assessing evidence about efficacy of combination therapy is whether the study was conducted in preclinical models or in patients. Hypothermia has shown clear neuroprotective effects in animal studies, but not sure in clinical trials. Hypothermia is induced differently in preclinical animal models and in patients and hypothermia complications such as shivering and infections are generally ignored in the laboratory but can pose substantial problems in the clinic. At the very least, the studies reviewed here make clear that hypothermia induction is feasible in stroke patients, though the optimal conditions still need to be explored.

This review also illustrates the broad range of neuroprotective treatments that have shown promise in preclinical studies, but have not yet been evaluated in the clinic or have failed to show success in patients. So far, only tissue plasminogen activator, intra-arterial recanalization, caffeinol and decompressive hemicraniectomy have been combined with hypothermia in stroke patients. Much more work remains to be done, which should take into account the heterogeneity of human stroke.

## References

[1] Reith J, Jorgensen HS, Pedersen PM, Nakayama H, Raaschou HO, Jeppesen LL and Olsen TS. (1996). Body temperature in acute stroke: relation to stroke severity, infarct size, mortality, and outcome. Lancet, 8999: 422-5. 10.1016/s0140-6736(96)90008-2 8618482

[2] Nagel S, Papadakis M, Hoyte L and Buchan AM. (2008). Therapeutic hypothermia in experimental models of focal and global cerebral ischemia and intracerebral hemorrhage. Expert Rev Neurother, 8: 1255-68. 18671669 10.1586/14737175.8.8.1255

[3] Jong YK, Midori AY. (2015). Hypothermia for treatment of stroke. Brain Circulation, 1: 14-25.

[4] Clark DL, Penner M, Orellana-Jordan IM and Colbourne F. (2008). Comparison of 12, 24 and 48 h of systemic hypothermia on outcome after permanent focal ischemia in rat. Exp Neurol, 2: 386-92. 10.1016/j.expneurol.2008.04.016 18538766

[5] Krieger DW and Yenari MA. (2004). Therapeutic hypothermia for acute ischemic stroke: what do laboratory studies teach us? Stroke, 6: 1482-9. 10.1161/01.STR.0000126118.44249.5c 15073396

[6] Maier CM, Ahern K, Cheng ML, Lee JE, Yenari MA and Steinberg GK. (1998). Optimal depth and duration of mild hypothermia in a focal model of transient cerebral ischemia: effects on neurologic outcome, infarct size, apoptosis, and inflammation. Stroke, 10: 2171-80. 10.1161/01.str.29.10.2171 9756600

[7] Ridenour TR, Warner DS, Todd MM and McAllister AC. (1992). Mild hypothermia reduces infarct size resulting from temporary but not permanent focal ischemia in rats. Stroke, 5: 733-8. 10.1161/01.str.23.5.733 1579972

[8] Polderman KH and Herold I. (2009). Therapeutic hypothermia and controlled normothermia in the intensive care unit: practical considerations, side effects, and cooling methods. Crit Care Med, 3: 1101-20. 10.1097/CCM.0b013e3181962ad5 19237924

[9] Bernard SA and Buist M. (2003). Induced hypothermia in critical care medicine: a review. Crit Care Med, 7: 2041-51. 10.1097/01.CCM.0000069731.18472.61 12847402

[10] Frazzini VI, Winfree CJ, Choudhri HF, Prestigiacomo CJ and Solomon RA. (1994). Mild hypothermia and MK-801 have similar but not additive degrees of cerebroprotection in the rat permanent focal ischemia model. Neurosurgery, 6: 1040-5; discussion 1045-6. 10.1227/00006123-199406000-00013 7916129

[11] Green EJ, Pazos AJ, Dietrich WD, McCabe PM, Schneiderman N, Lin B, et al (1995). Combined postischemic hypothermia and delayed MK-801 treatment attenuates neurobehavioral deficits associated with transient global ischemia in rats. Brain Res, 1-2: 145-52. 10.1016/0006-8993(95)01034-1 8846069

[12] Dietrich WD, Lin B, Globus MY, Green EJ, Ginsberg MD and Busto R. (1995). Effect of delayed MK-801 (dizocilpine) treatment with or without immediate postischemic hypothermia on chronic neuronal survival after global forebrain ischemia in rats. J Cereb Blood Flow Metab, 6: 960-8. 10.1038/jcbfm.1995.122 7593357

[13] Corbett D, Evans S, Thomas C, Wang D and Jonas RA. (1990). MK-801 reduced cerebral ischemic injury by inducing hypothermia. Brain Res, 2: 300-4. 10.1016/0006-8993(90)91424-f 2162711

[14] Hattori H and Wasterlain CG. (1991). Hypothermia does not explain MK-801 neuroprotection in a rat model of neonatal hypoxic-ischemic encephalopathy. Neurology, 2 (Pt 1): 330. 10.1212/wnl.41.2_part_1.330 1992389

[15] Valera E, Sanchez-Martin FJ, Ferrer-Montiel AV, Messeguer A and Merino JM. (2008). NMDA-induced neuroprotection in hippocampal neurons is mediated through the protein kinase A and CREB (cAMP-response element-binding protein) pathway. Neurochem Int, 5: 148-54. 10.1016/j.neuint.2008.07.007 18694792

[16] Haberny KA, Paule MG, Scallet AC, Sistare FD, Lester DS, Hanig JP and Slikker W Jr., (2002). Ontogeny of the N-methyl-D-aspartate (NMDA) receptor system and susceptibility to neurotoxicity. Toxicol Sci, 1: 9-17. 10.1093/toxsci/68.1.9 12075105

[17] Ikonomidou C, Bosch F, Miksa M, Bittigau P, Vockler J, Dikranian K, et al (1999). Blockade of NMDA receptors and apoptotic neurodegeneration in the developing brain. Science, 5398: 70-4. 10.1126/science.283.5398.70 9872743

[18] Shuaib A, Waqar T, Wishart T and Kanthan R. (1995). Post-ischemic therapy with CGS-19755 (alone or in combination with hypothermia) in gerbils. Neurosci Lett, 1-2: 87-90. 10.1016/0304-3940(95)11567-0 7659298

[19] Shuaib A, Ijaz S, Mazagri R and Senthilsevlvan A. (1993). CGS-19755 is neuroprotective during repetitive ischemia: this effect is significantly enhanced when combined with hypothermia. Neuroscience, 4: 915-20. 10.1016/0306-4522(93)90137-5 8284043

[20] Traynelis SF, Wollmuth LP, McBain CJ, Menniti FS, Vance KM, Ogden KK, et al (2010). Glutamate receptor ion channels: structure, regulation, and function. Pharmacol Rev, 3: 405-96. 10.1124/pr.109.002451 PMC2964903 20716669

[21] Zhu H, Meloni BP, Moore SR, Majda BT and Knuckey NW. (2004). Intravenous administration of magnesium is only neuroprotective following transient global ischemia when present with post-ischemic mild hypothermia. Brain Res, 1-2: 53-60. 10.1016/j.brainres.2004.03.073 15212991

[22] Campbell K, Meloni BP and Knuckey NW. (2008). Combined magnesium and mild hypothermia (35 degrees C) treatment reduces infarct volumes after permanent middle cerebral artery occlusion in the rat at 2 and 4, but not 6 h. Brain Res, 258-64. 18644354 10.1016/j.brainres.2008.06.110

[23] Song W, Wu YM, Ji Z, Ji YB, Wang SN and Pan SY. (2013). Intra-carotid cold magnesium sulfate infusion induces selective cerebral hypothermia and neuroprotection in rats with transient middle cerebral artery occlusion. Neurol Sci, 4: 479-86. 10.1007/s10072-012-1064-3 22466873

[24] Meloni BP, Cross JL, Brookes LM, Clark VW, Campbell K and Knuckey NW. (2013). FAST-Mag protocol with or without mild hypothermia (35 degrees C) does not improve outcome after permanent MCAO in rats. Magnes Res, 2: 67-73. 10.1684/mrh.2013.0340 23816810

[25] Nagel S, Su Y, Horstmann S, Heiland S, Gardner H, Koziol J, et al (2008). Minocycline and hypothermia for reperfusion injury after focal cerebral ischemia in the rat: effects on BBB breakdown and MMP expression in the acute and subacute phase. Brain Res, 198-206. 10.1016/j.brainres.2007.10.052 18031717

[26] Thoresen M, Satas S, Puka-Sundvall M, Whitelaw A, Hallstrom A, Loberg EM, et al (1997). Post-hypoxic hypothermia reduces cerebrocortical release of NO and excitotoxins. Neuroreport, 15: 3359-62. 10.1097/00001756-199710200-00033 9351672

[27] Tang XN, Liu L, Koike MA and Yenari MA. (2013). Mild hypothermia reduces tissue plasminogen activator-related hemorrhage and blood brain barrier disruption after experimental stroke. Ther Hypothermia Temp Manag, 2: 74-83. 10.1089/ther.2013.0010 PMC3684213 23781399

[28] Kallmunzer B, Schwab S and Kollmar R. (2012). Mild hypothermia of 34 degrees C reduces side effects of rt-PA treatment after thromboembolic stroke in rats. Exp Transl Stroke Med, 1: 3. 10.1186/2040-7378-4-3 PMC3320523 22397464

[29] Meden P, Overgaard K, Pedersen H and Boysen G. (1994). Effect of hypothermia and delayed thrombolysis in a rat embolic stroke model. Acta Neurol Scand, 2: 91-8. 10.1111/j.1600-0404.1994.tb02686.x 7801745

[30] Kollmar R, Henninger N, Bardutzky J, Schellinger PD, Schabitz WR and Schwab S. (2004). Combination therapy of moderate hypothermia and thrombolysis in experimental thromboembolic stroke--an MRI study. Exp Neurol, 1: 204-12. 10.1016/j.expneurol.2004.07.006 15473993

[31] Hemmen TM, Raman R, Guluma KZ, Meyer BC, Gomes JA, Cruz-Flores S, et al (2010). Intravenous thrombolysis plus hypothermia for acute treatment of ischemic stroke (ICTuS-L): final results. Stroke, 10: 2265-70. 10.1161/STROKEAHA.110.592295 PMC2947593 20724711

[32] Bi M, Ma Q, Zhang S, Li J, Zhang Y, Lin L, et al (2011). Local mild hypothermia with thrombolysis for acute ischemic stroke within a 6-h window. Clin Neurol Neurosurg, 9: 768-73. 10.1016/j.clineuro.2011.08.010 21893379

[33] Piironen K, Tiainen M, Mustanoja S, Kaukonen KM, Meretoja A, Tatlisumak T and Kaste M. (2014). Mild hypothermia after intravenous thrombolysis in patients with acute stroke: a randomized controlled trial. Stroke, 2: 486-91. 10.1161/STROKEAHA.113.003180 24436240

[34] Hong JM, Lee JS, Song HJ, Jeong HS, Choi HA and Lee K. (2014). Therapeutic hypothermia after recanalization in patients with acute ischemic stroke. Stroke, 1: 134-40. 10.1161/STROKEAHA.113.003143 24203846

[35] Ji X. (2015). Forward thinking in stroke treatment: Advances in cerebrovascular reperfusion and neurorehabilitation. Brain Circulation, 1: 1-2.

[36] Lapchak PA. (2015). Critical early thrombolytic and endovascular reperfusion therapy for acute ischemic stroke victims: a call for adjunct neuroprotection. Transl Stroke Res, 5: 345-54. 10.1007/s12975-015-0419-5 PMC4568436 26314402

[37] Chen J, Liu L, Zhang H, Geng X, Jiao L, Li G, et al (2016). Endovascular Hypothermia in Acute Ischemic Stroke: Pilot Study of Selective Intra-Arterial Cold Saline Infusion. Stroke, 7: 1933-5. 10.1161/STROKEAHA.116.012727 PMC4927369 27197848

[38] Lelekov-Boissard T, Chapuisat G, Boissel JP, Grenier E and Dronne MA. (2009). Exploration of beneficial and deleterious effects of inflammation in stroke: dynamics of inflammation cells. Philos Trans A Math Phys Eng Sci, 1908: 4699-716. 19884176 10.1098/rsta.2009.0184

[39] Yenari MA and Han HS. (2006). Influence of hypothermia on post-ischemic inflammation: role of nuclear factor kappa B (NFkappaB). Neurochem Int, 2: 164-9. 10.1016/j.neuint.2006.03.016 16750872

[40] Furuichi Y, Katsuta K, Maeda M, Ueyama N, Moriguchi A, Matsuoka N, et al (2003). Neuroprotective action of tacrolimus (FK506) in focal and global cerebral ischemia in rodents: dose dependency, therapeutic time window and long-term efficacy. Brain Res, 1-2: 137-45. 10.1016/s0006-8993(02)04151-3 12591130

[41] Noto T, Ishiye M, Furuich Y, Keida Y, Katsuta K, Moriguchi A, et al (2004). Neuroprotective effect of tacrolimus (FK506) on ischemic brain damage following permanent focal cerebral ischemia in the rat. Brain Res Mol Brain Res, 1: 30-8. 10.1016/j.molbrainres.2004.06.003 15337315

[42] Nito C, Kamiya T, Ueda M, Arii T and Katayama Y. (2004). Mild hypothermia enhances the neuroprotective effects of FK506 and expands its therapeutic window following transient focal ischemia in rats. Brain Res, 2: 179-85. 10.1016/j.brainres.2004.02.031 15145754

[43] Koistinaho M, Malm TM, Kettunen MI, Goldsteins G, Starckx S, Kauppinen RA, et al (2005). Minocycline protects against permanent cerebral ischemia in wild type but not in matrix metalloprotease-9-deficient mice. J Cereb Blood Flow Metab, 4: 460-7. 10.1038/sj.jcbfm.9600040 15674236

[44] Amin AR, Patel RN, Thakker GD, Lowenstein CJ, Attur MG and Abramson SB. (1997). Post-transcriptional regulation of inducible nitric oxide synthase mRNA in murine macrophages by doxycycline and chemically modified tetracyclines. FEBS Lett, 2-3: 259-64. 10.1016/s0014-5793(97)00605-4 9237641

[45] Soliman S, Ishrat T, Fouda AY, Patel A, Pillai B and Fagan SC. (2015). Sequential Therapy with Minocycline and Candesartan Improves Long-Term Recovery After Experimental Stroke. Transl Stroke Res, 4: 309-22. 10.1007/s12975-015-0408-8 PMC4559761 26004281

[46] Chen M, Ona VO, Li M, Ferrante RJ, Fink KB, Zhu S, et al (2000). Minocycline inhibits caspase-1 and caspase-3 expression and delays mortality in a transgenic mouse model of Huntington disease. Nat Med, 7: 797-801. 10.1038/77528 10888929

[47] Jordan J, Fernandez-Gomez FJ, Ramos M, Ikuta I, Aguirre N and Galindo MF. (2007). Minocycline and cytoprotection: shedding new light on a shadowy controversy. Curr Drug Deliv, 3: 225-31. 10.2174/156720107781023938 17627496

[48] Wang CX, Yang T and Shuaib A. (2003). Effects of minocycline alone and in combination with mild hypothermia in embolic stroke. Brain Res, 1-2: 327-9. 10.1016/s0006-8993(02)04045-3 12560140

[49] Wang CX, Yang T, Noor R and Shuaib A. (2002). Delayed minocycline but not delayed mild hypothermia protects against embolic stroke. BMC Neurol, 2. 11960560 10.1186/1471-2377-2-2 PMC107740

[50] Rosenson RS. (2001). Pluripotential mechanisms of cardioprotection with HMG-CoA reductase inhibitor therapy. Am J Cardiovasc Drugs, 6: 411-20. 10.2165/00129784-200101060-00001 14728000

[51] Zhu J, Song W, Li L and Fan X. (2016). Endothelial nitric oxide synthase: a potential therapeutic target for cerebrovascular diseases. Mol Brain, 30. 27000187 10.1186/s13041-016-0211-9 PMC4802712

[52] Lee SH, Kim YH, Kim YJ and Yoon BW. (2008). Atorvastatin enhances hypothermia-induced neuroprotection after stroke. J Neurol Sci, 1-2: 64-8. 10.1016/j.jns.2008.07.029 18768189

[53] Chan PH. (1996). Role of oxidants in ischemic brain damage. Stroke, 6: 1124-9. 10.1161/01.str.27.6.1124 8650725

[54] Wenjun L, Shaohua Y. (2016). Targeting oxidative stress for the treatment of ischemic stroke: Upstream and downstream therapeutic strategies. Brain Circulation, 4: 153-163. 10.4103/2394-8108.195279 PMC6126224 30276293

[55] Takizawa Y, Miyazawa T, Nonoyama S, Goto Y and Itoh M. (2009). Edaravone inhibits DNA peroxidation and neuronal cell death in neonatal hypoxic-ischemic encephalopathy model rat. Pediatr Res, 6: 636-41. 10.1203/PDR.0b013e3181a16a9f 19247215

[56] Noor JI, Ueda Y, Ikeda T and Ikenoue T. (2007). Edaravone inhibits lipid peroxidation in neonatal hypoxic-ischemic rats: an in vivo microdialysis study. Neurosci Lett, 1: 5-9. 10.1016/j.neulet.2006.10.024 17280782

[57] Nito C, Kamiya T, Amemiya S, Katoh K and Katayama Y. (2003). The neuroprotective effect of a free radical scavenger and mild hypothermia following transient focal ischemia in rats. Acta Neurochir Suppl, 199-203. 14753435 10.1007/978-3-7091-0651-8_43

[58] Zhang X, Wang X, Zhang J, Huang X, Wei D, Lan W and Hu Z. (2016). Reduction of nitrous oxide emissions from partial nitrification process by using innovative carbon source (mannitol). Bioresour Technol, 789-95. 10.1016/j.biortech.2016.07.043 27423546

[59] Kazan S, Karasoy M, Baloglu H and Tuncer R. (1999). The effect of mild hypothermia, mannitol and insulin-induced hypoglycaemia on ischaemic infarct volume in the early period after permanent middle cerebral artery occlusion in the rat. Acta Neurochir (Wien), 9: 979-87. 10.1007/s007010050405 10526080

[60] Karibe H, Zarow GJ and Weinstein PR. (1995). Use of mild intraischemic hypothermia versus mannitol to reduce infarct size after temporary middle cerebral artery occlusion in rats. J Neurosurg, 1: 93-8. 10.3171/jns.1995.83.1.0093 7782857

[61] Davalos A and Secades J. (2011). Citicoline preclinical and clinical update 2009-2010. Stroke, 1 Suppl: S36-9. 10.1161/STROKEAHA.110.605568 21164116

[62] Secades JJ. (2011). Citicoline: pharmacological and clinical review, 2010 update. Rev Neurol, S1-S62. 21432836

[63] Weiss GB. (1995). Metabolism and actions of CDP-choline as an endogenous compound and administered exogenously as citicoline. Life Sci, 9: 637-60. 10.1016/0024-3205(94)00427-t 7869846

[64] Gutierrez-Fernandez M, Rodriguez-Frutos B, Fuentes B, Vallejo-Cremades MT, Alvarez-Grech J, Exposito-Alcaide M and Diez-Tejedor E. (2012). CDP-choline treatment induces brain plasticity markers expression in experimental animal stroke. Neurochem Int, 3: 310-7. 10.1016/j.neuint.2011.12.015 22226841

[65] Sahin S, Alkan T, Temel SG, Tureyen K, Tolunay S and Korfali E. (2010). Effects of citicoline used alone and in combination with mild hypothermia on apoptosis induced by focal cerebral ischemia in rats. J Clin Neurosci, 2: 227-31. 10.1016/j.jocn.2009.05.016 20036128

[66] Strong R, Grotta JC and Aronowski J. (2000). Combination of low dose ethanol and caffeine protects brain from damage produced by focal ischemia in rats. Neuropharmacology, 3: 515-22. 10.1016/s0028-3908(99)00156-2 10698017

[67] Chandler LJ, Sumners C and Crews FT. (1993). Ethanol inhibits NMDA receptor-mediated excitotoxicity in rat primary neuronal cultures. Alcohol Clin Exp Res, 1: 54-60. 10.1111/j.1530-0277.1993.tb00726.x 8383926

[68] Aronowski J, Strong R, Shirzadi A and Grotta JC. (2003). Ethanol plus caffeine (caffeinol) for treatment of ischemic stroke: preclinical experience. Stroke, 5: 1246-51. 10.1161/01.STR.0000068170.80517.B3 12690223

[69] Martin-Schild S, Hallevi H, Shaltoni H, Barreto AD, Gonzales NR, Aronowski J, et al (2009). Combined neuroprotective modalities coupled with thrombolysis in acute ischemic stroke: a pilot study of caffeinol and mild hypothermia. J Stroke Cerebrovasc Dis, 2: 86-96. 10.1016/j.jstrokecerebrovasdis.2008.09.015 PMC2673088 19251183

[70] Ford JM, Prozialeck WC and Hait WN. (1989). Structural features determining activity of phenothiazines and related drugs for inhibition of cell growth and reversal of multidrug resistance. Mol Pharmacol, 1: 105-15. 2563302

[71] Snyder SH, Banerjee SP, Yamamura HI and Greenberg D. (1974). Drugs, neurotransmitters, and schizophrenia. Science, 4143: 1243-53. 10.1126/science.184.4143.1243 17784215

[72] Liu S, Geng X, Forreider B, Xiao Y, Kong Q, Ding Y and Ji X. (2015). Enhanced beneficial effects of mild hypothermia by phenothiazine drugs in stroke therapy. Neurol Res, 5: 454-60. 10.1179/1743132815Y.0000000031 25819773

[73] Patel PM, Drummond JC, Cole DJ, Kelly PJ and Watson M. (1998). Isoflurane and pentobarbital reduce the frequency of transient ischemic depolarizations during focal ischemia in rats. Anesth Analg, 4: 773-80. 10.1097/00000539-199804000-00018 9539600

[74] Kimbro JR, Kelly PJ, Drummond JC, Cole DJ and Patel PM. (2000). Isoflurane and pentobarbital reduce AMPA toxicity in vivo in the rat cerebral cortex. Anesthesiology, 3: 806-12. 10.1097/00000542-200003000-00024 10719959

[75] Warner DS, Zhou JG, Ramani R and Todd MM. (1991). Reversible focal ischemia in the rat: effects of halothane, isoflurane, and methohexital anesthesia. J Cereb Blood Flow Metab, 5: 794-802. 10.1038/jcbfm.1991.137 1874810

[76] Smith DS, Rehncrona S and Siesjo BK. (1980). Barbiturates as protective agents in brain ischemia and as free radical scavengers in vitro. Acta Physiol Scand Suppl, 129-34. 6939303

[77] Westermaier T, Zausinger S, Baethmann A, Steiger HJ and Schmid-Elsaesser R. (2000). No additional neuroprotection provided by barbiturate-induced burst suppression under mild hypothermic conditions in rats subjected to reversible focal ischemia. J Neurosurg, 5: 835-44. 10.3171/jns.2000.93.5.0835 11059666

[78] Varathan S, Shibuta S, Shimizu T, Varathan V and Mashimo T. (2002). Hypothermia and thiopentone sodium: individual and combined neuroprotective effects on cortical cultures exposed to prolonged hypoxic episodes. J Neurosci Res, 3: 352-62. 10.1002/jnr.10237 12111866

[79] David HN, Haelewyn B, Rouillon C, Lecoq M, Chazalviel L, Apiou G, et al (2008). Neuroprotective effects of xenon: a therapeutic window of opportunity in rats subjected to transient cerebral ischemia. FASEB J, 4: 1275-86. 10.1096/fj.07-9420com 18024836

[80] Dickinson R, Peterson BK, Banks P, Simillis C, Martin JC, Valenzuela CA, et al (2007). Competitive inhibition at the glycine site of the N-methyl-D-aspartate receptor by the anesthetics xenon and isoflurane: evidence from molecular modeling and electrophysiology. Anesthesiology, 5: 756-67. 10.1097/01.anes.0000287061.77674.71 18073551

[81] Weber NC, Toma O, Wolter JI, Obal D, Mullenheim J, Preckel B and Schlack W. (2005). The noble gas xenon induces pharmacological preconditioning in the rat heart in vivo via induction of PKC-epsilon and p38 MAPK. Br J Pharmacol, 1: 123-32. 10.1038/sj.bjp.0706063 PMC1575984 15644876

[82] Liu X, Dingley J, Scull-Brown E and Thoresen M. (2015). Adding 5 h delayed xenon to delayed hypothermia treatment improves long-term function in neonatal rats surviving to adulthood. Pediatr Res, 6: 779-83. 10.1038/pr.2015.49 25760545

[83] Fries M, Brucken A, Cizen A, Westerkamp M, Lower C, Deike-Glindemann J, et al (2012). Combining xenon and mild therapeutic hypothermia preserves neurological function after prolonged cardiac arrest in pigs. Crit Care Med, 4: 1297-303. 10.1097/CCM.0b013e31823c8ce7 22425822

[84] Sheng SP, Lei B, James ML, Lascola CD, Venkatraman TN, Jung JY, et al (2012). Xenon neuroprotection in experimental stroke: interactions with hypothermia and intracerebral hemorrhage. Anesthesiology, 6: 1262-75. 10.1097/ALN.0b013e3182746b81 23143806

[85] Hoffman WE, Kochs E, Werner C, Thomas C and Albrecht RF. (1991). Dexmedetomidine improves neurologic outcome from incomplete ischemia in the rat. Reversal by the alpha 2-adrenergic antagonist atipamezole. Anesthesiology, 2: 328-32. 10.1097/00000542-199108000-00022 1677549

[86] Zeng X, Wang H, Xing X, Wang Q and Li W. (2016). Dexmedetomidine Protects against Transient Global Cerebral Ischemia/Reperfusion Induced Oxidative Stress and Inflammation in Diabetic Rats. PLoS One, 3: e0151620. 10.1371/journal.pone.0151620 PMC4794239 26982373

[87] Luo C, Yuan D, Yao W, Cai J, Zhou S, Zhang Y and Hei Z. (2015). Dexmedetomidine protects against apoptosis induced by hypoxia/reoxygenation through the inhibition of gap junctions in NRK-52E cells. Life Sci, 72-7. 10.1016/j.lfs.2014.12.009 25529146

[88] Sato K, Kimura T, Nishikawa T, Tobe Y and Masaki Y. (2010). Neuroprotective effects of a combination of dexmedetomidine and hypothermia after incomplete cerebral ischemia in rats. Acta Anaesthesiol Scand, 3: 377-82. 10.1111/j.1399-6576.2009.02139.x 19860751

[89] Wang C, Nguyen HN, Maguire JL and Perry DC. (2002). Role of intracellular calcium stores in cell death from oxygen-glucose deprivation in a neuronal cell line. J Cereb Blood Flow Metab, 2: 206-14. 10.1097/00004647-200202000-00008 11823718

[90] Li F, Hayashi T, Jin G, Deguchi K, Nagotani S, Nagano I, et al (2005). The protective effect of dantrolene on ischemic neuronal cell death is associated with reduced expression of endoplasmic reticulum stress markers. Brain Res, 1-2: 59-68. 10.1016/j.brainres.2005.04.058 15921666

[91] Hadad E, Cohen-Sivan Y, Heled Y and Epstein Y. (2005). Clinical review: Treatment of heat stroke: should dantrolene be considered? Crit Care, 1: 86-91. 10.1186/cc2923 PMC1065088 15693989

[92] Muehlschlegel S and Sims JR. (2009). Dantrolene: mechanisms of neuroprotection and possible clinical applications in the neurointensive care unit. Neurocrit Care, 1: 103-15. 10.1007/s12028-008-9133-4 PMC2702250 18696266

[93] Xu SY, Hu FY, Ren LJ, Chen L, Zhou ZQ, Zhang XJ and Li WP. (2015). Dantrolene enhances the protective effect of hypothermia on cerebral cortex neurons. Neural Regen Res, 8: 1279-85. 10.4103/1673-5374.162761 PMC4590241 26487856

[94] Chen C, Cui H, Li Z, Wang R and Zhou C. (2013). Normobaric oxygen for cerebral ischemic injury. Neural Regen Res, 31: 2885-94. 10.3969/j.issn.1673-5374.2013.31.001 PMC4146175 25206609

[95] Tang X, Liu KJ, Ramu J, Chen Q, Li T and Liu W. (2010). Inhibition of gp91(phox) contributes towards normobaric hyperoxia afforded neuroprotection in focal cerebral ischemia. Brain Res, 174-80. 10.1016/j.brainres.2010.05.082 PMC3073844 20547141

[96] Kim HY, Singhal AB and Lo EH. (2005). Normobaric hyperoxia extends the reperfusion window in focal cerebral ischemia. Ann Neurol, 4: 571-5. 10.1002/ana.20430 15786465

[97] Liu W, Sood R, Chen Q, Sakoglu U, Hendren J, Cetin O, et al (2008). Normobaric hyperoxia inhibits NADPH oxidase-mediated matrix metalloproteinase-9 induction in cerebral microvessels in experimental stroke. J Neurochem, 5: 1196-205. 10.1111/j.1471-4159.2008.05664.x PMC2582595 18786175

[98] Cai L, Thibodeau A, Peng C, Ji X, Rastogi R, Xin R, et al (2016). Combination therapy of normobaric oxygen with hypothermia or ethanol modulates pyruvate dehydrogenase complex in thromboembolic cerebral ischemia. J Neurosci Res, 8: 749-58. 10.1002/jnr.23740 27027410

[99] Cai L, Stevenson J, Geng X, Peng C, Ji X, Xin R, et al (2016). Combining Normobaric Oxygen with Ethanol or Hypothermia Prevents Brain Damage from Thromboembolic Stroke via PKC-Akt-NOX Modulation. Mol Neurobiol10.1007/s12035-016-9695-7 26820681

[100] Cai L, Stevenson J, Peng C, Xin R, Rastogi R, Liu K, et al (2016). Adjuvant therapies using normobaric oxygen with hypothermia or ethanol for reducing hyperglycolysis in thromboembolic cerebral ischemia. Neuroscience, 45-57. 10.1016/j.neuroscience.2016.01.010 26794589

[101] Weinstein PR, Anderson GG and Telles DA. (1987). Results of hyperbaric oxygen therapy during temporary middle cerebral artery occlusion in unanesthetized cats. Neurosurgery, 4: 518-24. 10.1227/00006123-198704000-00002 3587541

[102] Weinstein PR, Hameroff SR, Johnson PC and Anderson GG. (1986). Effect of hyperbaric oxygen therapy or dimethyl sulfoxide on cerebral ischemia in unanesthetized gerbils. Neurosurgery, 5: 528-32. 10.1227/00006123-198605000-00002 3713999

[103] Liu W, Khatibi N, Sridharan A and Zhang JH. (2011). Application of medical gases in the field of neurobiology. Medical gas research, 1: 13. 22146102 10.1186/2045-9912-1-13 PMC3231869

[104] Singhal AB, Ratai E, Benner T, Vangel M, Lee V, Koroshetz WJ, et al (2007). Magnetic resonance spectroscopy study of oxygen therapy in ischemic stroke. Stroke, 10: 2851-4. 10.1161/STROKEAHA.107.487280 17761914

[105] Wada K, Nishi D, Kitamura T, Ono K, Takahara T, Shirotani T and Shimizu A. (2006). Hyperbaric oxygenation therapy enhances the protective effect of moderate hypothermia against forebrain ischemia in the gerbil hippocampus. Undersea Hyperb Med, 6: 399-405. 17274309

[106] Minnerup J, Heidrich J, Wellmann J, Rogalewski A, Schneider A and Schabitz WR. (2008). Meta-analysis of the efficacy of granulocyte-colony stimulating factor in animal models of focal cerebral ischemia. Stroke, 6: 1855-61. 10.1161/STROKEAHA.107.506816 18403735

[107] Kong T, Choi JK, Park H, Choi BH, Snyder BJ, Bukhari S, et al (2009). Reduction in programmed cell death and improvement in functional outcome of transient focal cerebral ischemia after administration of granulocyte-macrophage colony-stimulating factor in rats. Laboratory investigation. J Neurosurg, 1: 155-63. 10.3171/2008.12.JNS08172 19361262

[108] Kovacic JC, Muller DW and Graham RM. (2007). Actions and therapeutic potential of G-CSF and GM-CSF in cardiovascular disease. J Mol Cell Cardiol, 1: 19-33. 10.1016/j.yjmcc.2006.10.001 17109881

[109] Buschmann IR, Busch HJ, Mies G and Hossmann KA. (2003). Therapeutic induction of arteriogenesis in hypoperfused rat brain via granulocyte-macrophage colony-stimulating factor. Circulation, 5: 610-5. 10.1161/01.CIR.0000074209.17561.99 12835229

[110] Schneider UC, Schilling L, Schroeck H, Nebe CT, Vajkoczy P and Woitzik J. (2007). Granulocyte-macrophage colony-stimulating factor-induced vessel growth restores cerebral blood supply after bilateral carotid artery occlusion. Stroke, 4: 1320-8. 10.1161/01.STR.0000259707.43496.71 17332468

[111] Todo K, Kitagawa K, Sasaki T, Omura-Matsuoka E, Terasaki Y, Oyama N, et al (2008). Granulocyte-macrophage colony-stimulating factor enhances leptomeningeal collateral growth induced by common carotid artery occlusion. Stroke, 6: 1875-82. 10.1161/STROKEAHA.107.503433 18388343

[112] Sugiyama Y, Yagita Y, Oyama N, Terasaki Y, Omura-Matsuoka E, Sasaki T and Kitagawa K. (2011). Granulocyte colony-stimulating factor enhances arteriogenesis and ameliorates cerebral damage in a mouse model of ischemic stroke. Stroke, 3: 770-5. 10.1161/STROKEAHA.110.597799 21257824

[113] Komine-Kobayashi M, Zhang N, Liu M, Tanaka R, Hara H, Osaka A, et al (2006). Neuroprotective effect of recombinant human granulocyte colony-stimulating factor in transient focal ischemia of mice. J Cereb Blood Flow Metab, 3: 402-13. 10.1038/sj.jcbfm.9600195 16049425

[114] Sehara Y, Hayashi T, Deguchi K, Zhang H, Tsuchiya A, Yamashita T, et al (2007). Decreased focal inflammatory response by G-CSF may improve stroke outcome after transient middle cerebral artery occlusion in rats. J Neurosci Res, 10: 2167-74. 10.1002/jnr.21341 17497673

[115] Ghahari L, Safari M, Joghataei MT, Mehdizadeh M and Soleimani M. (2014). Effect of combination therapy using hypothermia and granulocyte colony-stimulating factor in a rat transient middle cerebral artery occlusion model. Iran Biomed J, 4: 239-44. 10.6091/ibj.13852.2014 PMC4225064 25326023

[116] Jackson R, Tilokee EL, Latham N, Mount S, Rafatian G, Strydhorst J, et al (2015). Paracrine Engineering of Human Cardiac Stem Cells With Insulin-Like Growth Factor 1 Enhances Myocardial Repair. J Am Heart Assoc, 9: e002104. 10.1161/JAHA.115.002104 PMC4599498 26363004

[117] Bondanelli M, Ambrosio MR, Onofri A, Bergonzoni A, Lavezzi S, Zatelli MC, et al (2006). Predictive value of circulating insulin-like growth factor I levels in ischemic stroke outcome. J Clin Endocrinol Metab, 10: 3928-34. 10.1210/jc.2006-1040 16882751

[118] Aberg D, Jood K, Blomstrand C, Jern C, Nilsson M, Isgaard J and Aberg ND. (2011). Serum IGF-I levels correlate to improvement of functional outcome after ischemic stroke. J Clin Endocrinol Metab, 7: E1055-64. 10.1210/jc.2010-2802 21508132

[119] Lioutas VA, Alfaro-Martinez F, Bedoya F, Chung CC, Pimentel DA and Novak V. (2015). Intranasal Insulin and Insulin-Like Growth Factor 1 as Neuroprotectants in Acute Ischemic Stroke. Transl Stroke Res, 4: 264-75. 10.1007/s12975-015-0409-7 PMC4861046 26040423

[120] Lagina AT 3rd, Calo L, Deogracias M, Sanderson T, Kumar R, Wider J and Sullivan JM. (2013). Combination therapy with insulin-like growth factor-1 and hypothermia synergistically improves outcome after transient global brain ischemia in the rat. Acad Emerg Med, 4: 344-51. 10.1111/acem.12104 23701341

[121] George S, Bennet L, Weaver-Mikaere L, Fraser M, Bouwmans J, Mathai S, et al (2011). White matter protection with insulin-like growth factor 1 and hypothermia is not additive after severe reversible cerebral ischemia in term fetal sheep. Dev Neurosci, 3-4: 280-7. 10.1159/000329923 21822007

[122] Geral C, Angelova A and Lesieur S. (2013). From molecular to nanotechnology strategies for delivery of neurotrophins: emphasis on brain-derived neurotrophic factor (BDNF). Pharmaceutics, 1: 127-67. 10.3390/pharmaceutics5010127 PMC3834942 24300402

[123] Tsukahara T, Yonekawa Y, Tanaka K, Ohara O, Wantanabe S, Kimura T, et al (1994). The role of brain-derived neurotrophic factor in transient forebrain ischemia in the rat brain. Neurosurgery, 2: 323-31; discussion 331. 10.1227/00006123-199402000-00016 8177394

[124] Yamashita K, Wiessner C, Lindholm D, Thoenen H and Hossmann KA. (1997). Post-occlusion treatment with BDNF reduces infarct size in a model of permanent occlusion of the middle cerebral artery in rat. Metab Brain Dis, 4: 271-80. 10.1007/BF02674671 9475500

[125] Schabitz WR, Sommer C, Zoder W, Kiessling M, Schwaninger M and Schwab S. (2000). Intravenous brain-derived neurotrophic factor reduces infarct size and counterregulates Bax and Bcl-2 expression after temporary focal cerebral ischemia. Stroke, 9: 2212-7. 10.1161/01.str.31.9.2212 10978054

[126] Akaike A, Katsuki H, Kume T and Maeda T. (1999). Reactive oxygen species in NMDA receptor-mediated glutamate neurotoxicity. Parkinsonism Relat Disord, 4: 203-7. 10.1016/s1353-8020(99)00038-3 18591141

[127] Tremblay R, Hewitt K, Lesiuk H, Mealing G, Morley P and Durkin JP. (1999). Evidence that brain-derived neurotrophic factor neuroprotection is linked to its ability to reverse the NMDA-induced inactivation of protein kinase C in cortical neurons. J Neurochem, 1: 102-11. 10.1046/j.1471-4159.1999.0720102.x 9886060

[128] Berger C, Schabitz WR, Wolf M, Mueller H, Sommer C and Schwab S. (2004). Hypothermia and brain-derived neurotrophic factor reduce glutamate synergistically in acute stroke. Exp Neurol, 2: 305-12. 10.1016/j.expneurol.2003.10.008 14736512

[129] Sena E, Wheble P, Sandercock P and Macleod M. (2007). Systematic review and meta-analysis of the efficacy of tirilazad in experimental stroke. Stroke, 2: 388-94. 10.1161/01.STR.0000254462.75851.22 17204689

[130] Hellstrom HO, Wanhainen A, Valtysson J, Persson L and Hillered L. (1994). Effect of tirilazad mesylate given after permanent middle cerebral artery occlusion in rat. Acta Neurochir (Wien), 3-4: 188-92. 10.1007/BF01406503 7847162

[131] Beck T and Bielenberg GW. (1990). Failure of the lipid peroxidation inhibitor U74006F to improve neurological outcome after transient forebrain ischemia in the rat. Brain Res, 1-2: 336-8. 10.1016/0006-8993(90)91779-g 2282527

[132] Schmid-Elsaesser R, Zausinger S, Hungerhuber E, Baethmann A and Reulen HJ. (1999). Neuroprotective effects of combination therapy with tirilazad and magnesium in rats subjected to reversible focal cerebral ischemia. Neurosurgery, 1: 163-71; discussion 171-2. 10.1097/00006123-199901000-00100 9894977

[133] Schmid-Elsaesser R, Hungerhuber E, Zausinger S, Baethmann A and Reulen HJ. (1999). Combination drug therapy and mild hypothermia: a promising treatment strategy for reversible, focal cerebral ischemia. Stroke, 9: 1891-9. 10.1161/01.str.30.9.1891 10471442

[134] Zausinger S, Westermaier T, Plesnila N, Steiger HJ and Schmid-Elsaesser R. (2003). Neuroprotection in transient focal cerebral ischemia by combination drug therapy and mild hypothermia: comparison with customary therapeutic regimen. Stroke, 6: 1526-32. 10.1161/01.STR.0000070841.31224.29 12730554

[135] Zausinger S, Scholler K, Plesnila N and Schmid-Elsaesser R. (2003). Combination drug therapy and mild hypothermia after transient focal cerebral ischemia in rats. Stroke, 9: 2246-51. 10.1161/01.STR.0000083622.65684.21 12893947

[136] Belayev L, Liu Y, Zhao W, Busto R and Ginsberg MD. (2001). Human albumin therapy of acute ischemic stroke: marked neuroprotective efficacy at moderate doses and with a broad therapeutic window. Stroke, 2: 553-60. 10.1161/01.str.32.2.553 11157196

[137] Chen J, Fredrickson V, Ding Y, Cheng H, Wang N, Ling F and Ji X. (2013). Enhanced neuroprotection by local intra-arterial infusion of human albumin solution and local hypothermia. Stroke, 1: 260-2. 10.1161/STROKEAHA.112.675462 23192754

[138] Hacke W, Schwab S, Horn M, Spranger M, De Georgia M and von Kummer R. (1996). ’Malignant’ middle cerebral artery territory infarction: clinical course and prognostic signs. Arch Neurol, 4: 309-15. 10.1001/archneur.1996.00550040037012 8929152

[139] Wartenberg KE. (2012). Malignant middle cerebral artery infarction. Curr Opin Crit Care, 2: 152-63. 10.1097/MCC.0b013e32835075c5 22322256

[140] Schwab S, Georgiadis D, Berrouschot J, Schellinger PD, Graffagnino C and Mayer SA. (2001). Feasibility and safety of moderate hypothermia after massive hemispheric infarction. Stroke, 9: 2033-5. 10.1161/hs0901.095394 11546893

[141] Doerfler A, Schwab S, Hoffmann TT, Engelhorn T and Forsting M. (2001). Combination of decompressive craniectomy and mild hypothermia ameliorates infarction volume after permanent focal ischemia in rats. Stroke, 11: 2675-81. 10.1161/hs1101.098369 11692033

[142] Allahtavakoli M, Kahnouei MH, Rezazadeh H, Roohbakhsh A, Mahmoodi MH, Moghadam-Ahmadi A and Zarisfi M. (2014). Delayed combination therapy of local brain hypothermia and decompressive craniectomy on acute stroke outcome in rat. Iran J Basic Med Sci, 7: 476-82. PMC4242916 25429337

[143] Els T, Oehm E, Voigt S, Klisch J, Hetzel A and Kassubek J. (2006). Safety and therapeutical benefit of hemicraniectomy combined with mild hypothermia in comparison with hemicraniectomy alone in patients with malignant ischemic stroke. Cerebrovasc Dis, 1-2: 79-85. 10.1159/000090007 16330868

[144] Nagahara H, Vocero-Akbani AM, Snyder EL, Ho A, Latham DG, Lissy NA, et al (1998). Transduction of full-length TAT fusion proteins into mammalian cells: TAT-p27Kip1 induces cell migration. Nat Med, 12: 1449-52. 10.1038/4042 9846587

[145] Asoh S, Ohsawa I, Mori T, Katsura K, Hiraide T, Katayama Y, et al (2002). Protection against ischemic brain injury by protein therapeutics. Proc Natl Acad Sci U S A, 26: 17107-12. 10.1073/pnas.262460299 PMC139277 12475933

[146] Katsura K, Takahashi K, Asoh S, Watanabe M, Sakurazawa M, Ohsawa I, et al (2008). Combination therapy with transductive anti-death FNK protein and FK506 ameliorates brain damage with focal transient ischemia in rat. J Neurochem, 1: 258-70. 10.1111/j.0022-3042.2008.05360.x 18363825

[147] Sakurazawa M, Katsura K, Saito M, Asoh S, Ohta S and Katayama Y. (2012). Mild hypothermia enhanced the protective effect of protein therapy with transductive anti-death FNK protein using a rat focal transient cerebral ischemia model. Brain Res, 86-92. 10.1016/j.brainres.2011.10.041 22099262

[148] Yenari MA, Zhao H, Giffard RG, Sobel RA, Sapolsky RM and Steinberg GK. (2003). Gene therapy and hypothermia for stroke treatment. Ann N Y Acad Sci, 54-68; discussion 79-81. 10.1111/j.1749-6632.2003.tb07511.x 12853295

[149] Lawrence MS, McLaughlin JR, Sun GH, Ho DY, McIntosh L, Kunis DM, et al (1997). Herpes simplex viral vectors expressing Bcl-2 are neuroprotective when delivered after a stroke. J Cereb Blood Flow Metab, 7: 740-4. 10.1097/00004647-199707000-00003 9270490

[150] Zhao H, Yenari MA, Sapolsky RM and Steinberg GK. (2004). Mild postischemic hypothermia prolongs the time window for gene therapy by inhibiting cytochrome C release. Stroke, 2: 572-7. 10.1161/01.STR.0000110787.42083.58 14726551

